# Complex interplay of emotional availability with migration-related and sociodemographic factors: an exploratory study of mothers and their infants with a history of migration and exile in Germany

**DOI:** 10.3389/fpsyg.2026.1778280

**Published:** 2026-03-24

**Authors:** Lena Schestag, Patrick Meurs, Janina Mehner-Gentner, Amal Sarhan, Korinna Fritzemeyer

**Affiliations:** 1 Psychologische Hochschule Berlin, Berlin, Germany; 2 University of Kassel, Kassel, Germany; 3 Kindergesundheitshaus e.V., Berlin, Germany; 4 Sigmund-Freud-Institut, Frankfurt, Germany; 5 Katholieke Universiteit Leuven, Leuven, Belgium

**Keywords:** applied psychoanalysis, early childhood, emotional availability, migration, pre- peri- post migration, trauma transmission

## Abstract

**Background:**

Children born in Germany, who have a so-called migration background face great challenges. This is not due to their parents' history of migration itself, but rather due to the complex interplay of trauma, sociodemographic factors and numerous social barriers faced by children with an immigration background, which have an impact on their early development. While the effects of (acute) parental trauma, which often accompany flight and migration, on their children have been increasingly studied in recent decades, the effects of peri-and post-migration circumstances have rarely been investigated.

**Method:**

Our exploratory statistical analysis is based on data obtained during the formative evaluation of the early prevention project “Strong together!.” The analysis aims to investigate the emotional availability of migrated mothers in their interactions with their children, and the association between emotional availability, migratory circumstances and sociodemographic factors. To address the first research question, video recordings of 25 mother–child dyads were evaluated using the *Emotional Availability Scales* (EA Scales). Findings of our sample were compared with samples of migrated as well as non-migrated mother-child dyads from other studies. To answer the second question, non-parametric tests were conducted to examine the associations between emotional availability and various migration-related and sociodemographic factors.

**Results:**

On average, ratings on the *sensitivity* scale indicated that the observed mothers were “inconsistently sensitive” in the interaction with their child. This result appears to be similar to that found in migrant parents in other studies. Also, sensitivity was lower than observed in non-migrant samples. A higher – albeit subclinical – trauma-related symptom load was associated with more sensitive, more structured and less hostile interactions. Those who had fled war or persecution scored higher on the *child responsiveness* scale and, to a lesser extent, the *sensitivity* scale. In addition, women who had been in Germany for a shorter period of time were more sensitive and less hostile in their interactions with their children. Results highlight the necessity to offer prevention to children born to migrant mothers, independent of their self-report on trauma-related symptoms. Furthermore, they suggest that there is no straightforward association between migration-related stress and emotional availability.

## 1 Introduction

Today, there are more displaced people in the world than ever before. In Germany, the majority of people, who applied for asylum in 2023, came from four of the world-wide major conflict and post-conflict countries: Ukraine, Syria, Afghanistan and Iraq (UNHCR, 2024). Fleeing war or persecution is generally more stressful than migrating for economic, social, or individual reasons. This is due to the often life-threatening and violent situation in the country of origin, a hasty and rarely planned departure to an often uncertain destination as well as dangerous escape routes (Grinberg and Grinberg, 2016; Meurs, 2017; Parens, 2001; Varvin et al., 2022; Zimmermann, 2015). However, even planned migration poses numerous challenges, especially in the post-migration phase, including identity crises, acculturation stress, language barriers, experiences of racism and isolation, uncertain prospects of remaining in the country, cramped living conditions and barriers to accessing education, healthcare, and employment (Horst and Grabska, 2015; Nowak et al., 2023; Sluzki, 2010). Building secure and positive relationships after resettlement and building trust in the host society is described as crucial for overcoming these challenges and obstacles (Grinberg and Grinberg, 2016; Meurs, 2017; see also Keilson, 1979; Müller, 2017; Zimmermann, 2015). However, the ensuing mental structures are fragile and can be disturbed by renewed life challenges, such as becoming a parent.

The simultaneous experience of migration or flight, settling in and having a child combines two major life events that both independently from each other already increase personal vulnerability. Parenthood is closely tied to both conscious and unconscious cultural beliefs and practices that provide a stabilizing function. This stabilizing effect may be diminished or lost after resettling in a foreign culture (Moro et al., 2022; cf. Fritzemeyer, 2019). Conversely however, becoming a parent after resettlement can also have a positive impact. This can occur when parenthood is associated with feelings of self-efficacy and competence as well as a sense of future, which counteract feelings of helplessness and powerlessness experienced during war and flight (Leuzinger-Bohleber and Fritzemeyer, 2016; Kanal and Rottmann, 2021; Moro et al., 2022). The emotional reactions of parents to the new situation, as well as to potentially traumatic experiences and migration-related stressors are therefore of great importance to the child (Fraiberg, 1982, 2011; Fritzemeyer, 2016; Moré, 2013; von Overbeck Ottino, 2022). As (Freud and Burlingham 1943) observed during the bombings of London during the Second World War: “…it is more the mother's emotions that the children will have to live through than their own.” (p. 36). This can also be observed in children who have not experienced trauma themselves (Finger-Trescher, 2004; Fritzemeyer, 2019; Grubrich-Simitis, 1979; Lambert et al., 2014; Leuzinger-Bohleber and Fritzemeyer, 2016; Moré, 2013; Shachar-Dadon et al., 2017).

Not everyone who experiences traumatic events subsequently develops a mental disorder or persistent stress reactions (Bozorgmehr et al., 2016). With sufficient time, the establishment of stable relationships, and the regaining of external security, many people succeed in integrating their experiences into their life with neither serious nor long-lasting consequences for their mental development. However, infants and young children particularly depend on a sensitive and responsive caregiver to e.g. regulate states of tension, a main prerequisite for secure attachment, as well as emotional and cognitive development. They cannot “wait” for their parents to recover from their traumatic experiences at their own pace (cf. Fraiberg, 2011). (Khan 1963) described the mother in her role as a “protective shield,” an “auxiliary ego to support his [the childs”] immature and unstable ego functions (p. 290)” in the preverbal phase and coined the term cumulative trauma for continuous and repetitive breaches of this “protective shield” function. In the context of the Shoah, (Grubrich-Simitis 1979) postulated that the parents' extreme traumatic experiences can lead to cumulative trauma in the next generation, if the parents are unable to fulfill their “stimulus protection (p. 1007)” function. (Schechter et al. 2003) have moreover carefully described that traumatized mothers stabilize their own emotional regulation by shutting out emotional weary of their infants, which reminds them of their own trauma-associated helplessness. They found that traumatized mothers attribute intentional agonizing behavior to their infants, with mothers e.g., saying that their infants intentionally “annoyed' them by crying. (Belt et al. 2012) more recently summarized that the intimate bodily dialogue between mother and infant mediates past and present unprocessed and unintegrated experiences of loss and trauma onto the next generation. It can lead to withdrawal, intrusive behavior, hostility and role reversal as well as frightening and confusing behavior on the mother's side, all of which are risk factors for children to developing problematic i.e., disorganized attachment (cf. Belt et al., 2012; Madigan et al., 2006; Main and Hesse, 1990). However, these dynamics are often difficult to detect in early infancy. Therefore, they might not be diagnosed adequately and interventions come too late.

Over 30 years ago, (Biringen and Robinson 1991) operationalised emotional availability as the ability of a parent-child dyad to share an emotional connection for empirical research. Since then, the *Emotional Availability Scales* have been continuously developed (EA Scales; Biringen, 2008, 4th edition). The central assumption is that the child plays an active role in the interaction and must therefore be included in the observation in the same way as the caregiver. The EA Scales comprise four dimensions for adults: *sensitivity, structuring, nonintrusiveness* and *nonhostility*. For children, the dimensions are: *child responsiveness* and *child involvement*. All dimensions are assessed in relation to the child or caregiver on a scale from 0 to 7 (see Biringen, 2008, and the Methods section below). Numerous studies have examined the associations between emotional availability and various parent-, child-, and family-related factors, as well as socioeconomic factors (reviews in Biringen et al., 2014; Putnick et al., 2014). Consistently, correlations have been found between low emotional availability and low income (Chaudhuri et al., 2009; Han and Lee, 2010; Trupe et al., 2018), lower levels of formal education (Chaudhuri et al., 2009; Célia et al., 2018), and reduced social support (Trupe et al., 2018; Célia et al., 2018; MacMillan et al., 2021; Easterbrooks et al., 2005).

Emotional availability is also an affect-focused approach to understanding the impact of parental traumatic experiences on their children, which has been studied empirically in various contexts (Beebe et al., 1997; Biringen et al., 2014; Bretherton, 2000; Cohen and Shulman, 2019; MacMillan et al., 2021; Fritzemeyer et al., 2019; Fritzemeyer, 2016; Lebiger-Vogel et al., 2022; Scharpf et al., 2023). In a sample of refugee and asylum-seeking mothers in the Netherlands, (Van Ee et al. 2012) observed mother-child interactions with average EA scores between 4.06 and 5.00. These results indicate inconsistent emotional availability. Furthermore, a correlation between increased post-traumatic stress symptoms and lower scores on the *sensitivity, structuring*, and *nonhostility* scales was found. In a later study, Van Ee et al.'s (2016) observed that the correlation between parental attachment representation and individual *sensitivity* is stronger when higher PTSD symptoms are reported, though attachment representation was found to have no direct influence on PTSD symptoms. (Fritzemeyer et al. 2019) also found in their study of mothers who had fled to Germany to escape war or persecution and their children, that these were significantly less sensitive, structuring, and had a tendency to be more hostile (as measured with the EA Scales), than mothers who had migrated for social or economic reasons. This association was evident even in the behavior of the infants who were war refugees and showed a tendency to be less responsive and less engaged in interactions (see also Fritzemeyer et al., 2019). Here, mother-child interactions were examined at an earlier stage (children aged 2.5–5.5 months), whereas the children in the aforementioned studies were, on average, 26–28 months old.

In the scarce literature on the effects of migration on emotional availability in early mother-child interaction among migrated mothers, most, i.e. the ones mentioned above, have focused on the causes of migration as well as the extent and severity of traumatic experiences and trauma-related symptoms. However, there is a lack of research investigating the relevance of other stress factors during and after a potentially traumatic migration experience for emotional availability. In order to describe the complex dynamics of the various, closely interwoven phases before, during, and after acute traumatisation (Keilson 1979) coined the term “sequential traumatization.” His study of Jewish orphans after the Second World War showed that the severity of psychological consequences experienced in adulthood was more closely related to the stresses of the post-traumatic sequence (i.e. the post-war period, placement with a foster family, the return to a family of origin that was often traumatized, and sometimes multiple changes in between caregivers) than to the acute trauma (direct persecution, deportation, separation from parents, concentration camps, or hiding with foster parents). He also demonstrated that children who experienced trauma at a younger age exhibited a higher incidence of mental disorders in their further development than children who were older at the time of experiencing the trauma.

In the context of migrant and refugee families, these findings suggest that the post-migration phase is crucial for preventing adverse consequences of traumatic experiences. They furthermore suggest that it is of great importance to screen for new parents who are having difficulties to be emotionally available in the relationship with their child and to develop interventions that support these families (cf. Leuzinger-Bohleber and Lebiger-Vogel, 2016). Also, there needs to be a better understanding of the socio-economic situation of these families including the context-specific peri- and post-migration factors associated with increased difficulties establishing a responsive and sensitive parent–child relationship.

This study aims to explore the emotional availability in a sample of refugee and migrant mothers with infants and young children in Germany and the barely studied relationship between pre-, peri- and post-migration factors and emotional availability. An exploratory approach was chosen because there is a lack of empirical research investigating the associations between migration-related factors and emotional availability.

## 2 Method

The data collection took place in 2019 and 2020 as part of the formative evaluation of the early prevention project “Gemeinsam stark!” (“Strong Together!”), which was designed for refugee families with young children (see Schestag et al., 2021). Ethical approval for the analysis of the data for scientific purposes has been obtained from the Ethics Committee of the Psychologische Hochschule Berlin (PHB). This cross-sectional study is based on a non-representative and non-randomly selected sample.

### 2.1 Sample

The sample was recruited from mothers participating in the early prevention project “Strong Together!.” This programme consists of guided mother-child groups with roughly six regular participants in each group that meet once a week. Participation can last from pregnancy until the child turns four years old (Schestag et al., 2021). Mothers were informed about the prevention project by birth clinic staff, midwives or refugee camp staff. If they showed interest usually the group leaders made first contact by phone and a home visit. “Strong Together!” is a follow-up project to the prevention project for migrant mothers “FIRST STEPS” (Lebiger-Vogel et al., 2018; Leuzinger-Bohleber and Lebiger-Vogel, 2016) and is based on assumptions of psychoanalytic and attachment theory. The sample comprised 25 mother-child dyads who had fled or migrated to Germany from countries in the Middle East and North Africa (MENA region). Five dyads from sub-Saharan Africa and Asia were excluded from the present study to avoid even greater heterogeneity in cultural backgrounds, and to focus on the countries of origin from which most migrants to Germany come (cf. UNHCR, 2024).

### 2.2 Implementation

After informing the participants verbally and in writing, the practical project staff, who also led the weekly groups, obtained their written consent to participate. Participation was unpaid and choosing not to participate had no negative consequences for the mothers. Once consent had been obtained, the group leaders carried out the questionnaires and video recordings. For this purpose, they received training and ongoing support from the evaluation research staff.

### 2.3 Measurement instruments

#### 2.3.1 Socio-economic and migration-related factors

The IDeA Social Background Inventory (Körner and Betz, 2012) was used to record socio-economic and migration-related factors. Questions ranged from household size, sex of the child, income, and the mothers' level of education to the country of origin, religious affiliation, residence status, housing conditions, family network, the cause of migration, the route taken, and the circumstances of migration. To capture pre-migration stress, the item “cause of migration” was dichotomised to distinguish between women who fled war or persecution and those who migrated for economic, social, or individual reasons. Items used to record peri-migration stress were added up on a scale of 0 to 7. These include:

(1) opportunity to say goodbye to friends and family;
(2) duration of flight (fulfilled if longer than one or two days);
(3) longer interruptions to flight (more than seven days);
(4) subjective stress experience greater than three (on a scale of one to five);
(5) separation from family members;
(6) use of more than two modes of transport;
(7) dependence on a smuggler.

Residence status was recorded on a four-point scale (4 = EU citizen with a permanent residence permit; 3 = temporary residence permit, e.g., asylum seeker, refugee, subsidiary protection or national deportation ban; 2 = ongoing proceedings; 1 = no permit/tolerated stay). Family network was recorded on a 3-point scale (3 = relatives in the same city; 2 = relatives in Germany; 1 = no relatives in Germany) and German language skills on a 5-point scale (1 = no language course completed; 2 = A1/A2 level; 3 = B1/B2 level; 4 = C1/C2 level; 5 = German as mother tongue). Six levels were introduced to find out the highest level of education (1 = no school attendance; 2 = school without qualification; 3 = compulsory schooling with qualification; 4 = secondary school with qualification; 5 = completed vocational training; 6 = completed university degree). For an overview of the migration-related factors examined, see Table 1.

**Table 1:** Overview of migration-related factors.

**Pre-migration factor**	**Peri-migration stress factors**	**Post-migration factors**
Cause of migration (0) Migration for economic, social or individual reasons (1) Flight from war or persecution	Sum of (1) opportunity to say goodbye to friends and family (2) duration of flight (3) longer interruptions to flight (4) subjective stress experience (5) separation from family members (6) use of more than two modes of transport (7) dependence on a smuggler	•Trauma-related symptoms (HTQ)^2^ •Residence status •German language skills •Family network in Germany •Years in Germany •Housing conditions^1^ •Current family income^1^
Traumatic events (HTQ)^2^		

^1^No further investigation due to lack of variance in the sample; ^2^Harvard Trauma Questionnaire (HTQ).

#### 2.3.2 Traumatic events and trauma-related symptoms

Parts II (Traumatic events) and IV (Trauma-related symptoms) of the Harvard Trauma Questionnaire (HTQ; Mollica et al., 1992) were used to assess pre- and peri-migration traumatic events as well as current trauma-related symptoms. The trauma-related symptoms were assessed using 16 items corresponding to PTSD symptoms in the DSM-IV (American Psychiatric Association, 2000), which were answered on a scale from 1 (not at all) to 4 (extremely). To record traumatic events, 17 items indicated whether the event was experienced personally, witnessed, or heard about. The items were first dichotomised for the calculations, with 1 meaning the experience was either personally experienced or witnessed and 0 meaning the experience was neither personally experienced nor witnessed (a comparable procedure to that in Mollica et al., 1992, and Vukčević et al., 2016). Subsequently, the total number of reported traumatic events was calculated for each participant. Although the HTQ is considered sufficiently valid and reliable for use in non-Western cultures (Mollica et al., 1992), the cut-off value of 2.5 proposed by the authors for identifying clinically significant symptoms in samples from other cultural groups should be interpreted with caution (Wind et al., 2017). In this sample, the internal consistency of the 16-item symptom scale was Cronbach's alpha = 0.96.

#### 2.3.3 Emotional availability

Emotional availability was assessed using the *Emotional Availability Scales* (EA Scales; Biringen, 2008, 4th edition), based on an average of 23-min video recordings [*M (SD)* = 23 (5) min] of mothers with their children. These videos were recorded between September 2019 and February 2020 by the leaders of the mother-child groups, either before or after group meetings or during home visits. The initial instruction to the mothers regarding the video recording was: “Interact with your child as you normally would.” Emotional availability is operationalised using six scales. Each scale consists of seven subscales relating to certain aspects of the construct and a direct score from 1 to 7, which provides a global assessment of the scale. Higher scores indicate a more favorable rating for the respective scale. S*ensitivity* measures the caregivers' affect as well as their perception of and responsiveness to the child's emotional expression. High scores represent optimal functioning, whereas mid-range scores indicate the presence of warmth but insufficient attunement to the child's signals and needs. Low scores reflect emotional detachment, whereas the lowest scores indicate disturbed, bizarre, or minimal interaction. The second scale, s*tructuring*, captures the caregiver's attempts to structure the interaction and provide a holding frame for the child. High scores indicate consistent guidance, limit-setting, and support while respecting the child's autonomy. Mid-range scores reflect structuring that is not well attuned to the child, for example, being excessive or inadequate in relation to the child's needs, whereas low scores indicate a lack of structuring. The capacity to provide a holding framework and to be available in the interaction without encroaching on the child's autonomy is assessed using the *nonintrusiveness* scale. Scores in the middle range reflect benign intrusiveness or overprotective tendencies, whereas the lowest scores represent unwarranted or physically intrusive behavior. The *nonhostility* scale measures the extent to which an adult shows negative affect or hostile behavior toward the child in general, ranging from a complete absence of hostility at the high end, through subtle or covert hostility at mid-range scores, to openly hostile behavior at the low end. *Child responsiveness* focuses on the child's affect and responsiveness to the caregiver. High scores are associated with an attentive, age-appropriate connection to the caregiver. Excessive closeness and compliance at the expense of the child's autonomy correspond to mid-range scores, whereas “underresponsiveness” or “overresponsiveness” indicate low scores. *Child involvement* refers to the child's initiative to engage the parent in their play and activities. Mid-range scores reflect behaviors in which the child somewhat over-engages the caregiver, such as creating stressful situations or having difficulty tolerating physical or emotional distance. Low scores indicate either overly excessive or insufficient involvement. For further analysis, only the direct scores of the EA Scales are considered due to content considerations and to enable better comparability with other studies (Biringen et al., 2014; Biringen, 2008). Authorized training and reliability testing are necessary in order to use the EA Scales. The ratings were carried out by the first and third authors. Rater agreement for the six direct score ratings of the EA Scales was determined using the intraclass correlation coefficient (two-way mixed effects model, absolute agreement, ^**^p < 0.001): *sensitivity* ICC = 0.83; *structuring* ICC = 0.90^**^; *nonintrusiveness* ICC = 0.86^**^; *nonhostility* ICC = 0.73^**^; *child responsiveness* ICC = 0.90^**^; *child involvement* ICC = 0.78^**^. This is within the range of good agreement for this sample (cf. Koo and Li, 2016). The *nonhostility* scale is an exception, as the raters only agreed moderately on this scale. Five mother–child dyads were excluded from the ICC calculation because one of the raters knew them personally.

### 2.4 Statistical analysis

Due to the small sample size and deviations from a normal distribution, non-parametric testing was used. Associations between sociodemographic and migration-related factors and EA Scales were examined using Spearman's rank correlations, Mann-Whitney-*U* tests, or median tests, depending on the scale level, the presence of variance and distribution equality. Where group sizes differed, statistical calculations were performed using Mann-Whitney-*U* tests, median tests, or Fisher's exact tests according to scale level, variance homogeneity (Levene's test), and distribution equality (K-S test for two samples). Due to the exploratory nature of the study, we did not correct for the alpha error (cf. Victor et al., 2010). Thus, specifying *p*-values does not imply statistical significance of the test. They should only be interpreted as an indication of a statistically noticeable trend. All reported *p*-values refer to a two-tailed test. To better understand associations with small *p*-values, Mann-Whitney-*U* tests, median tests, and Fisher's exact tests were calculated. All calculations were performed using IBM SPSS (version 29.0.0.0).

## 3 Results

### 3.1 Sociodemographic factors

At the time of the survey, the mothers were, on average, 31 years old and their children were 20 months old. All participants were married, lived in rented accommodation, and were at risk of poverty (see Statista, 2025). These factors were therefore not considered in the subsequent statistical analysis. On average, the mothers had attended school for ten years. The vast majority (88%) stated Sunni Islam as their religion. Detailed socio-demographic information can be found in Table 2.

**Table 2:** Sociodemographic characteristics of the total sample (*N* = 25).

**Variable**		**Total sample**
Mean age	Mothers (in years)	*M* (*SD*): 31.32 (6.05)
	Children (in months)	*M* (*SD*): 20.24 (12.25)
Sex of children		m: 48%, f: 52%
Household size		*M* (*SD*): 4.56 (1.08)
Family status	Married	100%
Monthly household income (*n* = 24)		*M* (*SD*): 1,573.13 (614.40)
Education	Mean duration of school education (in years)	*M* (*SD*): 10.66 (2.26)
Highest qualification	School without qualification	20%
	Compulsory schooling with qualification	28%
	Secondary schooling with qualification	48%
	Completed vocational training	20%
	Completed university degree	28%
Countries of origin	Syria	60%
	Iraq, Lebanon, Algeria	each 8%
	Afghanistan, Egypt, Palestine, Kuwait	each 4%
Religion	Muslim – sunni	88%
	Muslim – shiite Muslim – without specification Yazidi	each 4%

### 3.2 Migration-related factors

Around two-thirds (72%) of participants stated that they had fled war or persecution, while 28% cited economic, social, or individual reasons for migrating (multiple answers were possible and classification was based on whether war or persecution was indicated). On average, their flight was associated with three peri-migration stress factors [*M* (*SD*) = 2.92 (2.31)]. 40% traveled to Germany in just one or two days, while 60% of participants took more than a week (an average of 73 days, ranging from 12 to 540 days) to reach Germany. 40% used more than two modes of transport. Around half (48%) arrived in Germany without any major interruptions to their journey. 56% stated that they had paid a smuggler and experienced a high to very high level of stress during their journey, whereas 40% stated that they experienced little or no stress. 28% were unable to say goodbye to family and friends. None of the participants were separated from their husbands or children at the time of data collection. 72% of the participants had a temporary residence permit. 60% of the participants had completed a language course (level B1/B2: 32%; level A1/A2: 28%). To assess family networks, information was collected on the proximity of relatives' residence: 16% of participants had no relatives in Germany; 32% had relatives in Germany, but not in the same city; 52% had relatives living in the same city. More than half of the participants arrived in Germany in 2015 or 2016; 32% of participants arrived in Germany before 2015; 12% after 2016 [*M* (*SD*) = 5.2 (4.8)]. 60% were stating Syria as their country of origin. Other countries of origin mentioned included Iraq, Lebanon, and Algeria (see Table 2).

### 3.3 Traumatic events and trauma-related symptoms

On average, participants reported experiencing six traumatic events in the HTQ [*M* (*SD*) = 5.92 (4.02)]. The most frequently reported traumatic events were “combat situations and armed conflicts” (64%) and “near-death experiences” (60%), followed by “lack of food or water” (52%). The events “poor health without access to medical care,” “no roof over one's head,” “forced separation from family members,” and “serious injury” were indicated by 48% of participants. The mean symptom scale value was 1.57 (*SD* = 0.66), corresponding to a low average symptom burden. Nine of the 25 participants stated that they did not experience any trauma-related symptoms.

### 3.4 Emotional availability

The direct scores of the six EA Scales are in the middle range, with values between 3.93 and 4.79 (see Table 3). On the *sensitivity* scale, this range describes an interaction characterized by “apparent/inconsistent sensitivity.” On average, the mothers are also inconsistent in their structuring of the interaction. There are signs of benign intrusiveness, while hostility is predominantly concealed. On average, the children appear complicated in their responsiveness and involvement with their mothers. This score indicates that a connection is at hand, but not secure (Biringen, 2008). Higher scores on the *child responsiveness* scale are associated with higher scores on all parental EA Scales. However, higher scores on the *child involvement* scale are only associated with higher scores on the *nonintrusiveness* scale.

**Table 3:** Mean emotional availability ratings and Spearman's rank correlations [95% confidence intervals] of parent- and child-related scales (*N* = 25).

**EA scale**	**Min**.	**Max**.	***M***	***SD***	**1**	**2**	**3**	**4**
1 *Sensitivity*	2.75	5.75	4.14	0.90				
2 *Structuring*	2.25	6.00	3.99	0.91				
3 *Nonintrusiveness*	2.25	5.75	3.93	0.92				
4 *Nonhostility*	3.00	6.25	4.79	0.88				
5 *Child responsiveness*	2.75	6.25	4.25	1.16	0.83^**^ [0.65, 0.93]	0.65^**^ [0.33, 0.83]	0.59^**^ [0.24, 0.80]	0.64^**^ [0.32, 0.83]
6 *Child involvement*	2.00	5.75	4.02	1.07	0.39 [−0.02, 0.69]	0.36 [−0.05, 0.67]	0.58^**^ [0.23, 0.80]	0.27 [−0.15, 0.61]

^**^ *p* ≤ .01^*^; *p* ≤ .05; 95% CIs based on formula by Fieller, Hartley and Pearson.

### 3.5 Correlations between emotional availability and socio-demographic factors

Examining the associations with Spearman's rank correlation between the EA Scales and socio-demographic characteristics (mother's and child's age, child's sex, household size, and level of education) revealed a correlation between child's age and the *nonintrusiveness* scale (*r* = 0.49, *p* = 0.014, 95% CI [0.10, 0.74]). This indicates that the older the child, the less intrusive the mother's behavior tends to be. None of the other correlations with sociodemographic factors resulted in low *p-*values.

### 3.6 Correlations between emotional availability and migration-related factors

Spearman's rank correlations between the eight migration-related factors (cause for migration, traumatic events, trauma-related symptoms, peri-migration stress, residence status, German language skills, family network in Germany, and years since arrival in Germany; see Table 1) and the EA Scales revealed correlations between trauma-related symptoms and the EA Scales *sensitivity* (*r* = 0.53, *p* = 0.006, 95% CI [0.16, 0.77]), *structuring* (*r* = 0.57, *p* = 0.003, 95% CI [0.21, 0.79]) and *nonhostility* (*r* = 0.57, *p* = 0.002, 95% CI [0.24, 0.57]). Mothers who reported more symptoms showed more sensitive and structured, and less hostile behavior in their interaction with their children. Furthermore, the results suggest that participants who fled war or persecution tend to exhibit greater sensitivity (*r* = 0.40, *p* = 0.050, 95% CIs [−0.01, 0.69]). Also, their children are more responsive (*r* = 0.49, *p* = 0.014, 95% CIs [0.10, 0.74]) in their interactions with them. Furthermore, mothers who had come to Germany earlier scored lower on the *sensitivity* (*r* = −0.55, *p* = 0.004, 95% CIs [−0.84, −0.26]) and *nonhostility* (*r* = −0.50, *p* = 0.011, 95% CIs [−0.81, −0.19]) scale. None of the other examined migration-related factors correlated with the variables of the EA Scales (see Table 4).

**Table 4:** Spearman's rank correlations [95% confidence intervals] of EA scales and migration-related factors (*N* = 25).

**Migration-related factor**	**Min**.	**Max**.	**M**	**SD**	***Sensitivity***	***Structuring***	***Nonintrusiveness***	***Nonhostility***	***Child responsiveness***	***Child involvement***
Cause of migration^a^	0	1	0.72	0.46	0.40^*^ [−0.01, 0.69]	0.33 [−0.09, 0.65]	0.22 [−0.21, 0.57]	0.31 [−0.11, 0.64]	0.49^*^ [0.10, 0.74]	0.18 [−0.24, 0.55]
Peri-migration stress factors	0	6	2.92	2.31	0.25 [−0.18, 0.59]	0.15 [−0.28, 0.52]	0.01 [−0.40, 0.42]	0.26 [−0.16, 0.60]	0.24 [−0.18, 0.59]	0.00 [−0.41, 41]
Traumatic events	0	13	5.92	4.02	−0.27 [−0.15, 0.61]	0.26 [−0.16, 0.60]	−0.18 [−0.55, 0.24]	0.22 [−0.21, 0.57]	0.30 [−0.12, 0.63]	0.02 [−0.39, 0.43]
Trauma-related symptoms	1.00	3.31	1.57	0.66	0.53^**^ [0.16, 0.77]	0.57^**^ [0.21, 0.79]	−0.05 [−0.45, 0.36]	0.59^**^ [0.24, 0.80]	0.32 [−0.09, 0.65]	−0.13 [−0.51, 0.29]
Residence status	3	4	3.28	0.46	−0.29 [−0.61, 0.14]	−0.06 [−0.46, 0.35]	−0.10 [−0.49, 0.32]	−0.36 [−0.67, 0.06]	−0.25 [−0.59, 0.18]	0.15 [−0.27, 0.52]
German language skills	1	3	1.92	0.86	−0.01 [−0.41, 0.40]	−0.01 [−0.41, 0.40]	0.11 [−0.31, 0.50]	0.09 [−0.33, 0.48]	0.01 [−0.40, 0.41]	0.12 [−0.30, 0.50]
Family network in Germany	1	5	3.36	1.50	−0.05 [−0.45, 0.36]	0.02 [−0.39, 0.43]	−0.09 [−0.48, 0.33]	−0.12 [−0.50, 0.30]	−0.03 [−0.43, 0.38]	0.21 [−0.21, 0.57]
Years in Germany	1	9 (26^b^)	5.20	4.79	−0.55^**^ [−0.78, −0.19]	−0.33 [−0.65, 0.09]	−0.08 [−0.47, 0.34]	−0.50^*^ [−0.75, −0.12]	−0.38 [−0.68, 0.03]	−0.09 [−0.48, 0.33]

^**^ *p* ≤ .01^*^; *p* ≤ .05; 95% CIs based on formula by Fieller, Hartley and Pearson; ^a^flight from war or persecution = 1, migration for economic, social or individual reasons = 0; ^b^the outlier has no decisive influence on size and *p*-values of the correlations: r(years in Germany, Sensitivity) = 0.53^**^, r(years in Germany, Nonhostility) = −0.43^*^.

### 3.7 Further statistical analysis

To elaborate on the Spearman's rank correlations with small *p*-values between the EA Scales *sensitivity, structuring* and *nonhostility* and trauma-related symptoms, as well as *sensitivity* and *child responsiveness* and the cause of migration, Mann-Whitney-*U* tests and, where necessary due to violations of assumptions, median tests were performed (see Table 5). In order to better understand the correlation between EA scores and trauma-related symptoms, the sample was divided into two groups based on reported symptoms: no indication of symptoms vs. indication of symptoms. Group comparisons revealed differences with small *p*-values depending on the indication of trauma-related symptoms for *sensitivity* (3.50 vs. 4.50), *structuring* (3.36 vs. 4.34), and *nonhostility* (4.14 vs. 5.16). The difference of approximately one scale point indicates that participants who did not report trauma-related symptoms tend to fall on the *sensitivity* scale within the range of a detached interaction with some aspects of inconsistently sensitive behavior, whereas the other group falls within the higher “inconsistently/apparently sensitive” range. On the *structuring* scale, participants who reported trauma-related symptoms were, on average, in the higher “inconsistently structuring” range, while the other group was in the “partially non structuring” range. On the *nonhostility* scale, the difference means classification in the “covert hostility” range or the “hostility largely absent” range. Examining group differences between participants depending on the cause of migration also yielded a small *p*-value: on the *sensitivity* scale, the value of 3.64 in the subgroup, who migrated for economic, social or individual reasons indicates, as above, detached interactions with some aspects of inconsistently sensitive behavior, while the higher value of 4.33 in the subgroup, who escaped war or persecution indicates an inconsistently sensitive interaction. The difference of one scale point between the refugee subgroup and the migrant subgroup (4.56 vs. 3.46) means - in terms of content - a classification to different ranges of the *child responsiveness* scale, even if no low *p*-value was achieved in the median test.

**Table 5:** Mean emotional availability ratings based on reported trauma-associated symptoms or cause of migration.

**EA scale**	**Test statistic**	**No indication of trauma-related symptoms in the HTQ (*n* = 9)**	**Indication of trauma-related symptoms in the HTQ (*n* = 16)**
*Sensitivity*		*M* (*SD*): 3.50 (0.73)	*M* (*SD*): 4.50 (0.79)
	*Mann-Whitney U test*	*U* = 121.000, *p* = 0.004	
*Structuring*		*M* (*SD*): 3.36 (0.83)	*M* (*SD*): 4.34 (0.77)
	Median test	Median = 4.00, *p* = 0.040	
*Nonhostility*		*M* (*SD*): 4.14 (0.69)	*M* (*SD*): 5.16 (0.77)
	Median test	Median = 4.75, *p* = 0.011	
		**Flight from war or persecution (*****n*** = **18)**	**Migration for economic, social or individual reasons (*****n*** = **7)**
*Sensitivity*		*M* (*SD*): 4.33 (0.80)	*M* (*SD*): 3.64 (0.80)
	*Mann-Whitney U test*	*U* = 95.000, *p* = 0.055	
*Child responsiveness*		*M* (*SD*): 4.56 (1.06)	*M* (*SD*): 3.46 (1.02)
	Median test	Median = 4.00, *p* = 0.073	

Specification of p-values for exact significance; variance homogeneity (Levene's test) given for all comparisons; due to the unequal distribution of the structuring, nonhostility, and child responsiveness scales in the respective groups, the median test was used. HTQ (Harvard Trauma Questionnaire).

In order to describe the groups of mothers who fled war or persecution or migrated for other reasons in a more differentiated manner, Table 6 presents socio-demographic and migration-related factors for the two groups separately. Participants with a history of flight or persecution reported experiencing more traumatic events (7 vs. 2) and peri-migration stress factors (4 vs. none). They were also more likely to have temporary residence status and less likely to have completed a German language course. 78% of refugee mothers came from Syria, whereas only 14% of migrant mothers stated Syria as their country of origin. On average, mothers who migrated for economic, social, or individual reasons were six years older and had lived in Germany for almost a year and a half longer. No striking differences were found between the two groups in terms of the trauma-related symptoms, family network, level of education, household size, or the age and sex of the child.

**Table 6:** Sociodemographic and migration-related aspects based on the cause of migration.

**Factor**	**Flight from war or persecution (*n* = 18)**	**Migration for economic, social or individual reasons (*n* = 7)**
Traumatic events (HTQ)	7.56 (3.26) >Median: 9, ≤ Median: 9	1.71 (2.43) >Median: 0, ≤ Median: 7
	Median test: Median = 7, exact significance *p* = 0.027	
Migration-related stress factors	3.94 (1.83) >Median: 12, ≤ Median: 6	0.29 (0.76) >Median: 0, ≤ Median: 7
	Median test: Median = 3, exact significance *p* = 0.005	
Residence status	89% temporary 11% permanent	29% temporary 71% permanent
	Fisher's exact test: Coefficient of contingency *C* = 0.52, *p* = 0.007	
German language skills	50% no course completed 33% A1/A2 level 17% B1/B2 level	14% no course completed 14% A1/A2 level 71% B1/B2 level
	*Mann-Whitney U test*: *U* = 27.000, *p* = 0.029	
Age of mother	29.44 (5.47) >Median: 5, ≤ Median: 13	36.14 (4.89) >Median: 6, ≤ Median: 1
	Median test: Median = 31, exact significance *p* = 0.021	
Years in Germany	4.83 (5.41) >Median: 2, ≤ Median: 16	6.14 (2.67) >Median: 6 ≤ Median: 1
	Median test: Median = 4, exact significance *p* = 0.001	
Country of origin Syria	78% Syria 22% other country of the MENA region	14% Syria 86% other country of the MENA region
	Fisher's exact test: Coefficient of contingency *C* = 0.50, *p* = 0.007	

*Mann-Whitney U tests*: Specification of *p*-values for exact significance; country of origin Syria = 1, other country of origin = 0.

## 4 Discussion

On all EA Scales the mother-child interactions examined were on average in the middle range (cf. Biringen, 2008). Higher responsiveness of the children, but not the involvement of the adult by the child, was associated with higher scores on the four parental EA Scales. A higher, but still subclinical, symptom burden was associated with more sensitive, better structured and less hostile behavior. Compared to migration for social, economic, or individual reasons, fleeing war or persecution was associated with greater responsiveness of children toward their mothers and, as a trend, with more sensitive behavior of mothers toward their children. The association, between aspects of emotional availability and trauma-related symptoms, appears to be stronger than that with the cause of migration. However, it should be noted that this interpretation of the associations in our sample is not based on statistical evidence, but purely on descriptive indications. Mothers who had been in Germany for a longer period of time were less sensitive and more hostile in their interactions with their children. The group of mothers who had fled war or persecution reported more traumatic events and peri-migration stress factors during their flight than the migrant mothers. In addition, the mothers who had fled war or persecution were more likely to come from Syria, had been in Germany for a shorter period of time, had an uncertain residence status, and were less likely to have completed a German language course.

Emotional availability in this sample thus does not appear to differ from that of mothers who sought asylum in the Netherlands and were treated at the psychosocial center for traumatized persons (Van Ee et al., 2012). Since the 4th edition of the EA Scales has been used predominantly in clinical or high-risk samples (see Biringen et al., 2022), there is a lack of low-risk comparison samples. In a non-refugee control sample of an Italian study (Salvatori et al., 2016), all scale values for parents and children were in the emotionally available range (scale values 5 to 7), as was the case in the low-risk sample of another Italian study, in which, however, only the four parental scales of emotional availability are reported (Frigerio et al., 2019). The results regarding emotional availability in our sample indicate that the mothers and their children have a clear need for support compared to low-risk mother-child dyads, although the self-reported trauma-related symptom burden is in the subclinical range - in contrast to the sample analyzed by (Van Ee et al. 2012).

One hypothesis why mothers, who reported no symptoms at all fall within the range of the *sensitivity* scale that is characterized by emotionally distant or brusque interaction (Biringen, 2008) could be that they may have isolated, repressed, denied or isolated their affects. It could be possible that this reflects what Grubrich-Simitis describes in the context of the extreme trauma of the Holocaust as “apathy and affect paralysis,” accompanied by an “inability to relate to people in any way other than dutifully” (1979, p. 999, translated by author). In addition, the aforementioned defense mechanisms could make it difficult to symbolize and become aware of trauma-related symptoms (Grubrich-Simitis, 1979). Another reason for reporting no or fewer symptoms despite psychological distress may be stigma associated with mental health problems (Ciftci et al., 2013) or a feeling of gratitude that prevents people from reporting psychological distress (cf. Fritzemeyer, 2016). Finally, empirical studies show that symptoms are often underestimated in self-reports by patients with PTSD (Beutler-Traktovenko et al., 2020; McKinnon et al., 2016). In our sample, mothers who reported subclinical symptoms were more sensitive in their interactions with their children. It is possible that these participants are better able to tolerate and symbolize painful feelings and memories and establish a trusting relationship with their familiar group leaders, despite their traumatic experiences. (Lev-Wiesel and Amir 2003) found a positive correlation between the ability to report experiences during the Second World War and trauma coping. Against this background, the complementary use of observational instruments such as the EA Scales is of great importance for the diagnosis of trauma consequences. This is particularly true in the area of prevention, where psychological distress and the need for intervention may not yet be as apparent as in the sample of refugees recruited at the Dutch National Center for the Treatment of Trauma as a Result of War, Persecution and Violence (Van Ee et al., 2012, 2016).

Mothers who fled war or persecution reported more traumatic events, greater peri-migration stress, more frequent uncertain residence status, and lower German language skills. However, fleeing war or persecution is, in our sample, associated with higher, albeit still suboptimal, emotional availability. This shows that there is no simple association between the cause of migration, the extent of traumatisation, and emotional availability. The result contradicts the findings of (Fritzemeyer et al. 2019), who point to an opposite correlation among participants in the project “FIRST STEPS” for migrant and refugee families. Various post-migration factors among the planned migration subgroup provide a possible explanation for their comparatively lower emotional availability. In this sample, migration for economic, social, or individual reasons is not only associated with an earlier arrival in Germany, but also with older mothers and greater diversity in countries of origin. In addition, even this group reports an average of two traumatic events. The observed affective distancing and brusqueness in mother-child interaction could be the effects of resignation and hopelessness that have increased over the years. The decisive influence of the phase after acute traumatisation on the processing of experiences has been demonstrated in various contexts (Becker, 2014; Keilson, 1979; Zimmermann, 2015). It stands to reason that this phase can also be highly significant for processing planned migration and resettlement experiences (Nowak et al., 2023; Sluzki, 2010). Migration is often associated with high expectations of improved living conditions (Grinberg and Grinberg, 2016). For women, and especially mothers, who are primarily occupied with childcare in the early years, the risk of social isolation is high (Leuzinger-Bohleber and Fritzemeyer, 2016; Sluzki, 2010). However, the consequences of this may only become apparent years later.

The hope for a better future may also seem increasingly unattainable due to the difficult social and political developments in Germany: experiences of social exclusion and racism, barriers in the education and health care systems, difficulties in finding work and housing, and the rise of right-wing extremist parties. In the long term, this could lead to more stress in everyday life, which in turn can have a negative impact on parental sensitivity (Chaudhuri et al., 2009; Van IJzendoorn et al., 2008). Participants who have only recently arrived in Germany and fled war or persecution may be even less aware of this, as the relief of having escaped an acute life-threatening situation still predominates (cf. phase of overcompensation, Sluzki, 2010). In addition, the consequences of potentially traumatic experiences are often delayed (cf. Bergmann, 1996, cited in Bohleber, 2000), which could also be a reason for the direct correlation between lower emotional availability and length of stay in Germany. Without knowing of the future challenges in exile, one's own immediate experience of competence and effectiveness in motherhood could provide support as an important resource during this phase of migration. Syrian women in exile also seem to experience a strong sense of self-efficacy and agency in relation to their motherhood (Kanal and Rottmann, 2021).

Another hypothesis for this result could be that the participants in this sample who fled war or persecution predominantly come from a similar context, namely the civil war in Syria. Processing traumatic events may cause less emotional distress in this sub-sample because of the shared experience of war, flight, and arrival. Belonging to a greater group from the same cultural context could have a protective effect on mental well-being and thus on mother-child relation. In contrast, individual migration experience could be more stressful (cf. Moro et al., 2022; Parens, 2001). The sub-sample of mothers who fled war or persecution, as studied by (Fritzemeyer et al. 2019), shows greater heterogeneity in terms of countries of origin. This also might be a reason why the family network of mothers who have fled war or persecution not differs greatly from that of mothers who have migrated for other reasons. Social support and stable, trusting relationships are often cited as the most important resources for processing traumatic experiences (cf. Csef, 2024; Kanal and Rottmann, 2021; Renner et al., 2020; Zbidat et al., 2020) and can have a positive effect on emotional availability (MacMillan et al., 2021; Trupe et al., 2018).

### 4.1 Limitations

As a result of the sample selection, the results cannot be generalized to other groups of refugee mothers. Mothers who participate in a prevention project may have a higher awareness of the importance of the mother-child relationship, which can lead to an overestimation of emotional availability. The small sample size, especially when performing multiple tests, also means that effects cannot be investigated with the necessary power and outliers have a greater influence on the results despite non-parametric testing. The confidence intervals are correspondingly large.

Questionnaires can only capture what is accessible to consciousness and may miss experiences that are repressed or associated with shame. Response behavior can also be biased by social desirability. In addition, questionnaire surveys can often provide a very limited picture of the complex and multi-layered migrant biographies. A case in point is the story of a project participant who survived a war in her country of origin and only migrated years later for social reasons (Schestag et al., 2022). In another case, it is known that the family was separated during their flight and remained apart for over two years, but at the time of the survey, the family reunion had already taken place.

Biringen points out that, ideally, scoring of the EA Scales should be carried out by raters from the same cultural background or with the aid of translation. Even though maternal sensitivity was originally conceptualized in a transcultural context (Ainsworth, 1967) and no clinically relevant differences were found between different Western cultures, its manifestation can vary interculturally (Putnick et al., 2014). Studies from the MENA region are lacking. Yet most of those seeking protection in Germany currently come from this region (UNHCR, 2024). At the same time, the EA Scales have often been used interculturally and under conditions where verbal understanding was hardly possible (e.g., Van Ee et al., 2012). Focusing on non-verbal behavior is legitimate, especially in early mother-child interaction (see https://emotionalavailability.com/best-practices/researchers/). Nevertheless, an observation situation in an intercultural context can lead to distortions related to the defense against anxiety-inducing aspects in countertransference (cf. Devereux, 1984). Also, internal racism of White, German researchers observing participants from a different culture (Davids, 2019) may produce misinterpretations. For example, unconscious aggressive feelings or feelings of guilt can influence assessments.

Finally, due to the small sample size, this may be a chance finding. It is essential that studies with larger samples investigate these associations.

### 4.2 Implications for practice

For practice, it is of central importance to recognize that supposedly objectifiable (external) stressors, like the cause of migration, or post-migration stressors, have only limited significance for emotional availability in early mother-child interaction. In addition, symptoms may only become apparent after a long period of time, when sufficiently safe external conditions have been restored (cf. Bergmann, 1996). It is therefore prudent to define the target groups for prevention offers quite broadly. In our practical project, for example, we would have excluded mother-child dyads who clearly need support if we had focused only on mothers who had fled war or persecution.

Sometimes irritations, like an early withdrawal by the child or a slightly hostile affect by the parent, are difficult to detect (cf. Khan, 1963). In early prevention, it is therefore important to pay attention to subtle signals from parents and children (Fraiberg, 2011; Moro et al., 2022). As the results show, children react promptly to less sensitive behavior on the part of their parents and appear less responsive in interactions even at a very young age. Observation tools, like the EA Scales, are a promising approach to be able to focus on less obvious irritations in interaction. “As [a] tool to ‘take the temperature' of relationships” (Biringen et al., 2014, p.114), they are relatively easy to use after training and certification due to their manageable time requirements, relative freedom of language, and flexibility in terms of setting.

Furthermore, there is evidence, that professional psychoanalytically informed prevention with an individual approach not only reaches migrant mothers better than usual care, but also fosters emotional availability in migrant mothers (Lebiger-Vogel et al., 2022). However, in some cases, the focus an early mother-child relationship might fall short. Further along in the post-migration phase, different concepts might be needed that place emphasis on the individual developmental needs and resources of mothers in order to counteract processes of resignation. Resignation processes might lead to decreasing emotional availability over time, as the association between sensitivity and length of stay in Germany in this study suggests. Continuous, secure, and trusting relationships with others are crucial for coping with traumatic and stressful experiences, especially in the phase following acute traumatisation. (Keilson 1979) already observed this in his study of Jewish war orphans, and it remains valid to this day (cf. Csef, 2024). To provide migrant and refugee families with secure and long-lasting relationships, long-term and continuous support services with staff continuity are needed in practical projects. Furthermore, different prevention programs from a single source would help to stay closely connected to participants, who are considered hard to reach, even when prevention requirements change over time. For example, we made the experience that mothers, who already participated in a mother-child group were eager to participate in a parenting school programme, which usually has difficulties reaching migrant families, as it was integrated in the groups where the familiar group leaders were also present. Another example is the successful establishment of a grief support group, which was well received primarily because it was set up in the same premises and with familiar staff. This contradicts the funding system in Germany, which favors model projects and often makes it difficult to secure continued funding for well-established projects. Also, in the face of recent crises and wars, social and political interest and thus support for (former) refugees is steadily declining.

### 4.3 Conclusion and further research

The results show how complex and multifaceted the migration and refugee histories in our sample are. Although not representative in its scope, it seems likely though that the complex histories in this sample provide valuable insights into a variety of migration and flight biographies. The subjective significance of traumatic events and migration-related stress factors, as well as individual resilience and resources, seem to play a major role in the development of trauma-related symptoms as well as the lasting restoration of mental health. As our results suggest, there is no direct association of traumatic experiences with the development of symptoms. Furthermore, the complex effects of these factors on emotional availability show that there is no simple dose-response relationship and that a broad, overarching concept of trauma and its intergenerational consequences falls short in the context of individual migration and flight biographies. Nevertheless, it becomes clear that migrant and refugee families need support to build emotionally available relationships with their children after resettlement. An approach is needed that takes into account each individual with their history, challenges and resources.

The challenges faced by migrant and refugee mothers in the post-migration phase must be included by future studies. Whether, in addition to the years spent in Germany, other post-migration stressors are associated with emotional availability remains unclear and needs to be further investigated with larger samples. Also, long-term studies would be beneficial in order to better understand developmental trajectories. The significant psychological impact of parental stress on children is undisputed. In addition to global ratings such as the EA Scales (Biringen, 2008), other promising approaches for identifying and describing the interpersonal mechanisms of transmitting trauma-related experiences among migrant and refugee families could be behavior-oriented concepts like the analysis of micro-interactions (cf. Stern, 2006) or early defense reactions (Fraiberg, 1982). These are also helpful tools for practical staff in order to reliably detect the often difficult-to-see, potentially problematic irritations in early interaction between migrant parents and their children, thus to be able to offer appropriate interventions in a timely manner. To this end, it is also necessary to investigate whether and to what extent the ratings of researchers from the same cultural background as the parent-child dyad differ from those of researchers from a different cultural background.

## Data Availability

The data analyzed in this study is subject to the following licenses/restrictions: The dataset for this article is not publicly available due to concerns regarding participant anonymity. Requests to access these datasets should be directed to Lena Schestag, l.schestag@phb.de.
